# Supplementary material for: Germline-targeting HIV Envelope SOSIP immunization more frequently elicits broadly-neutralizing antibody precursor responses in infant compared to juvenile rhesus macaques
Source: bioRxiv. 2025 May 30:2025.05.27.656273. Preprint. [Version 1] doi: 10.1101/2025.05.27.656273 (PMC12154809; doi:10.1101/2025.05.27.656273)
Supplement: 1 [file NIHPP2025.05.27.656273V1-supplement-1.pdf]

BG505 GT1.1

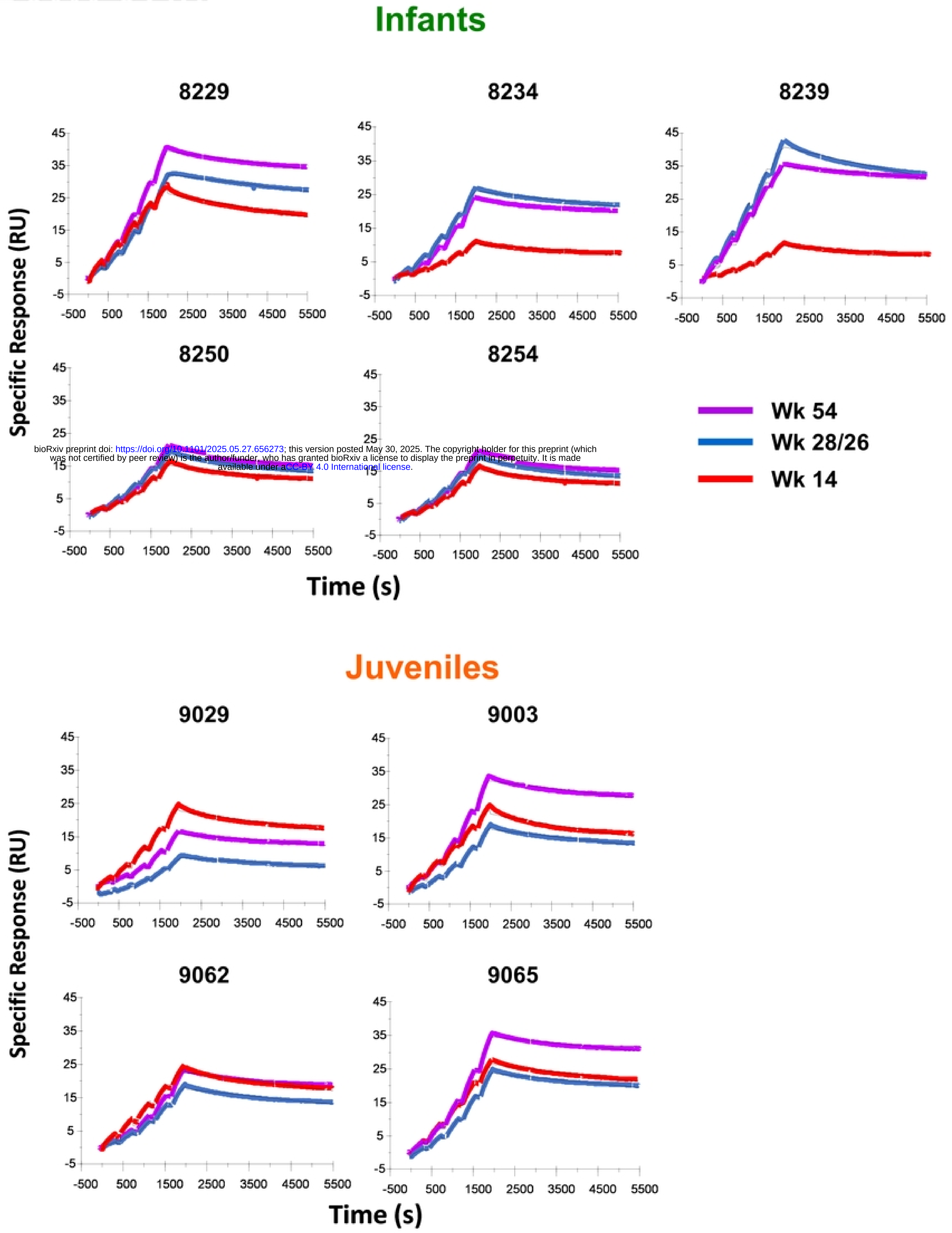

bioRxiv preprint doi: https://doi.org/10.1101/2025.05.27.656273; this version posted May 30, 2025. The copyright holder for this preprint (which was not certified by peer review) is the author/funder, who has granted bioRxiv a license to display the preprint in perpetuity. It is made available under aCC-BY 4.0 International license.

Supp Fig 1

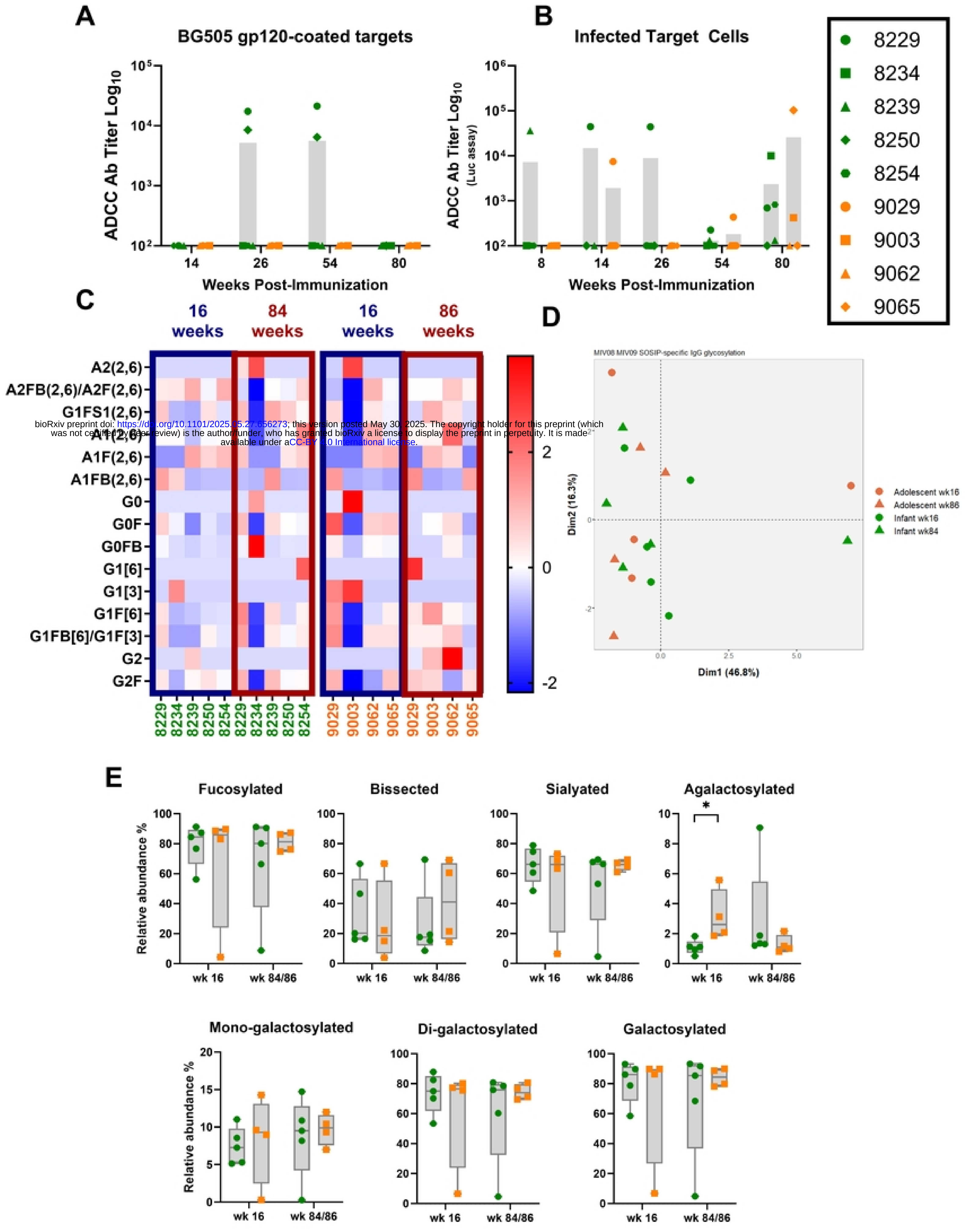

bioRxiv preprint doi: https://doi.org/10.1101/2025.05.27.656273; this version posted May 30, 2025. The copyright holder for this preprint (which was not certified by peer review) is the author/funder, who has granted bioRxiv a license to display the preprint in perpetuity. It is made available under aCC-BY 4.0 International license.

Supp Fig 2

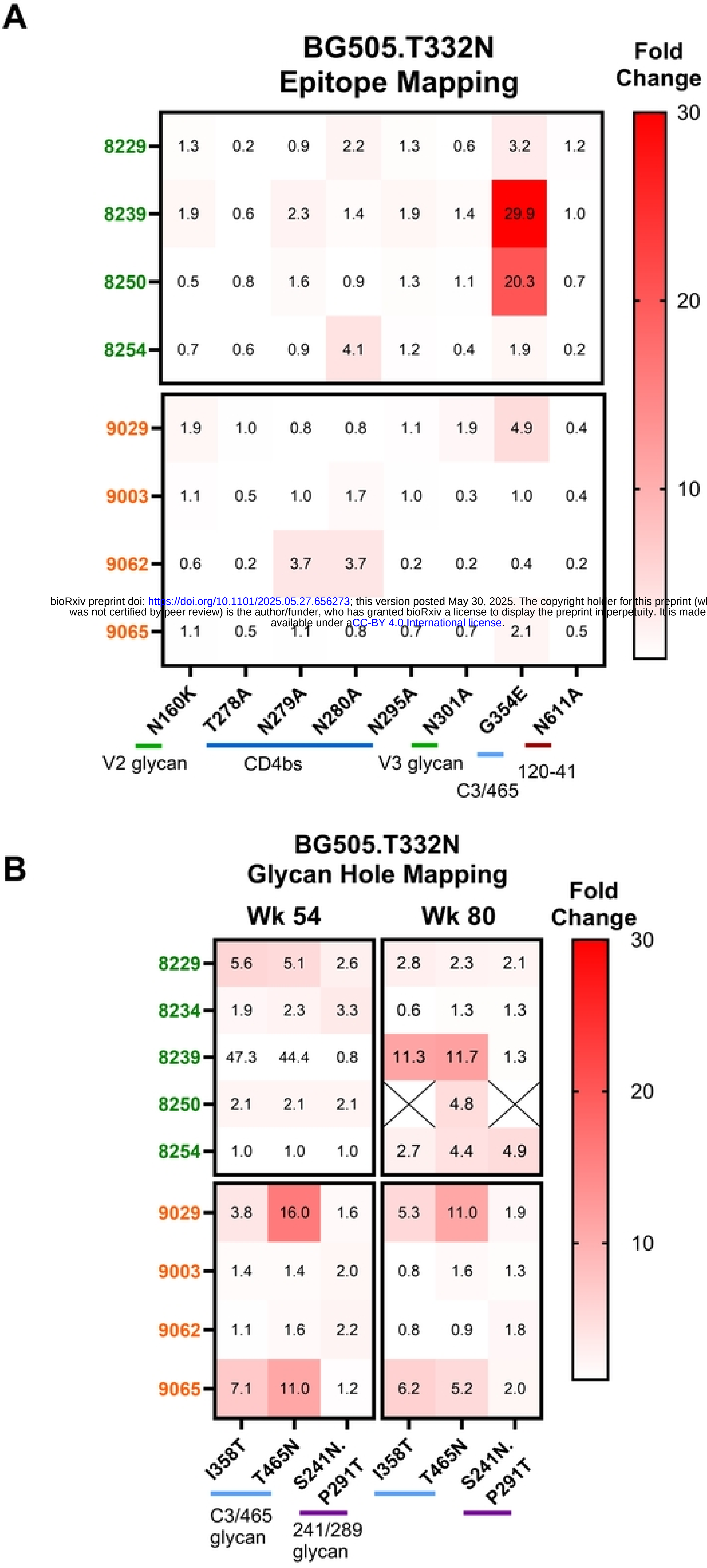

bioRxiv preprint doi: https://doi.org/10.1101/2025.05.27.656273; this version posted May 30, 2025. The copyright holder for this preprint (which was not certified by peer review) is the author/funder, who has granted bioRxiv a license to display the preprint in perpetuity. It is made available under aCC-BY 4.0 International license.

Supp Fig 3

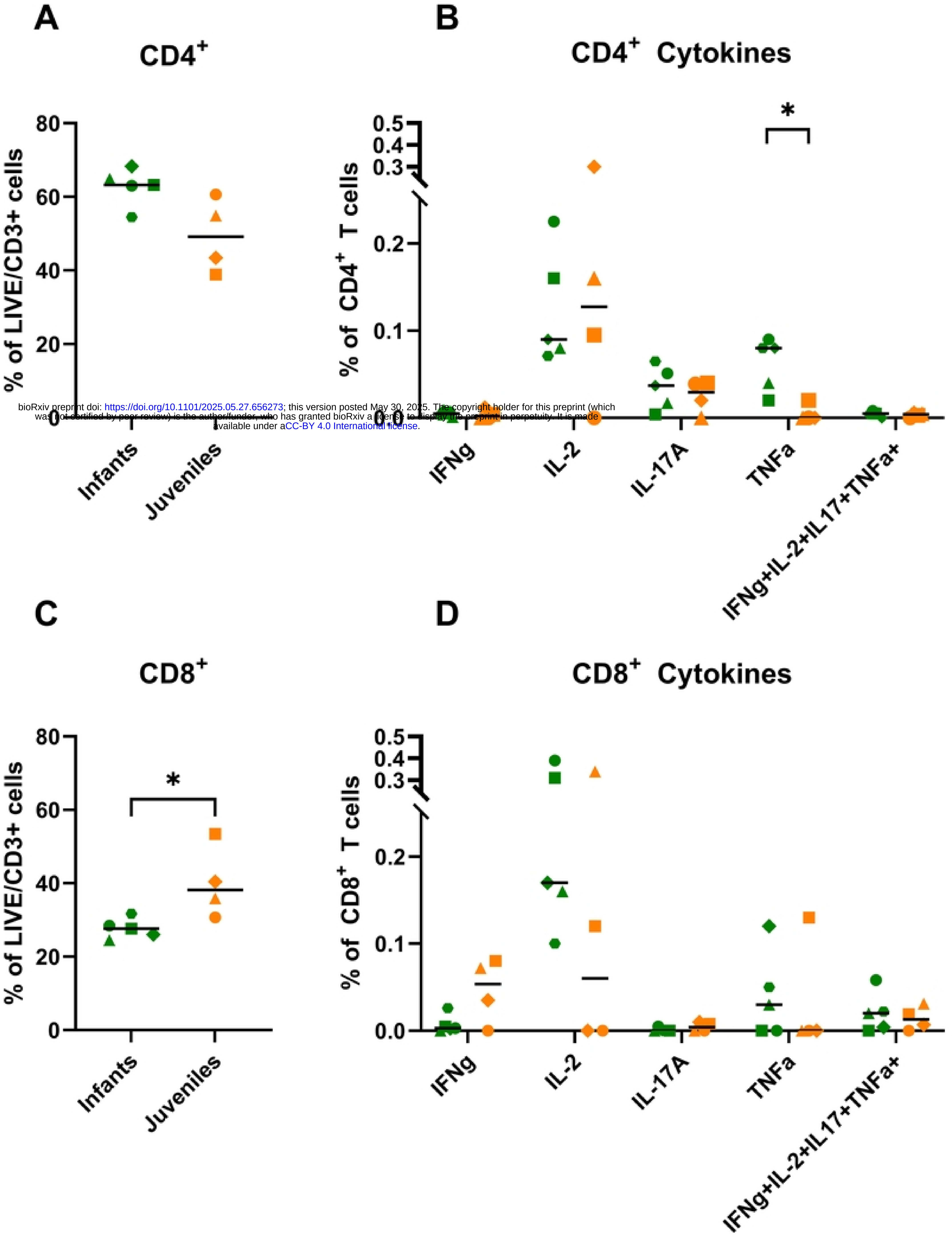

bioRxiv preprint doi: https://doi.org/10.1101/2025.05.27.656273; this version posted May 30, 2025. The copyright holder for this preprint (which was not certified by peer review) is the author/funder, who has granted bioRxiv a license to display the preprint in perpetuity. It is made available under aCC-BY 4.0 International license.

Supp Fig 5

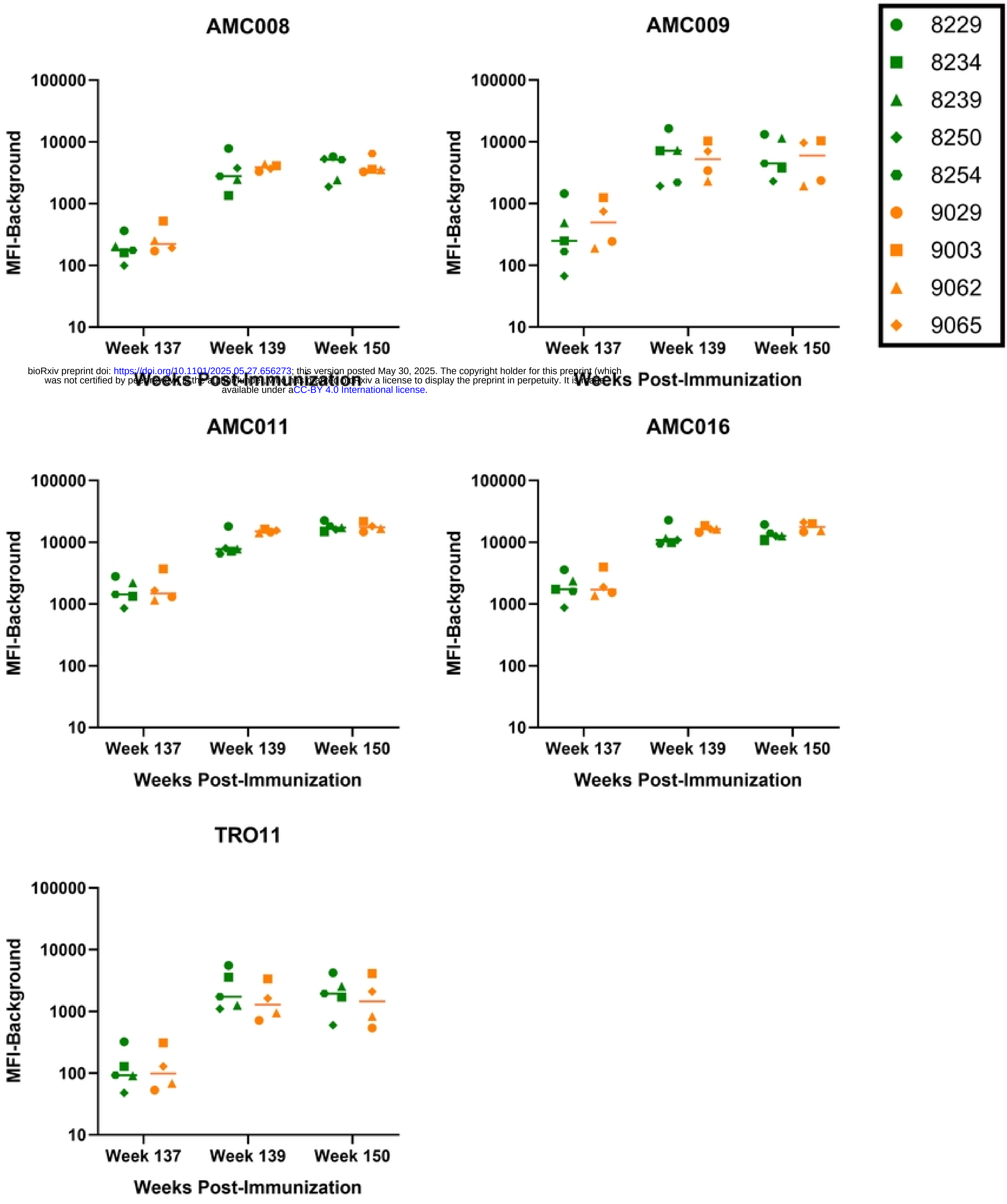

bioRxiv preprint doi: https://doi.org/10.1101/2025.05.27.656273; this version posted May 30, 2025. The copyright holder for this preprint (which was not certified by peer review) is the author/funder, who has granted bioRxiv a license to display the preprint in perpetuity. It is made available under aCC-BY 4.0 International license.

Supp Fig 6

bioRxiv preprint doi: https://doi.org/10.1101/2025.05.27.656273; this version posted May 30, 2025. The copyright holder for this preprint (which was not certified by peer review) is the author/funder, who has granted bioRxiv a license to display the preprint in perpetuity. It is made available under aCC-BY 4.0 International license.

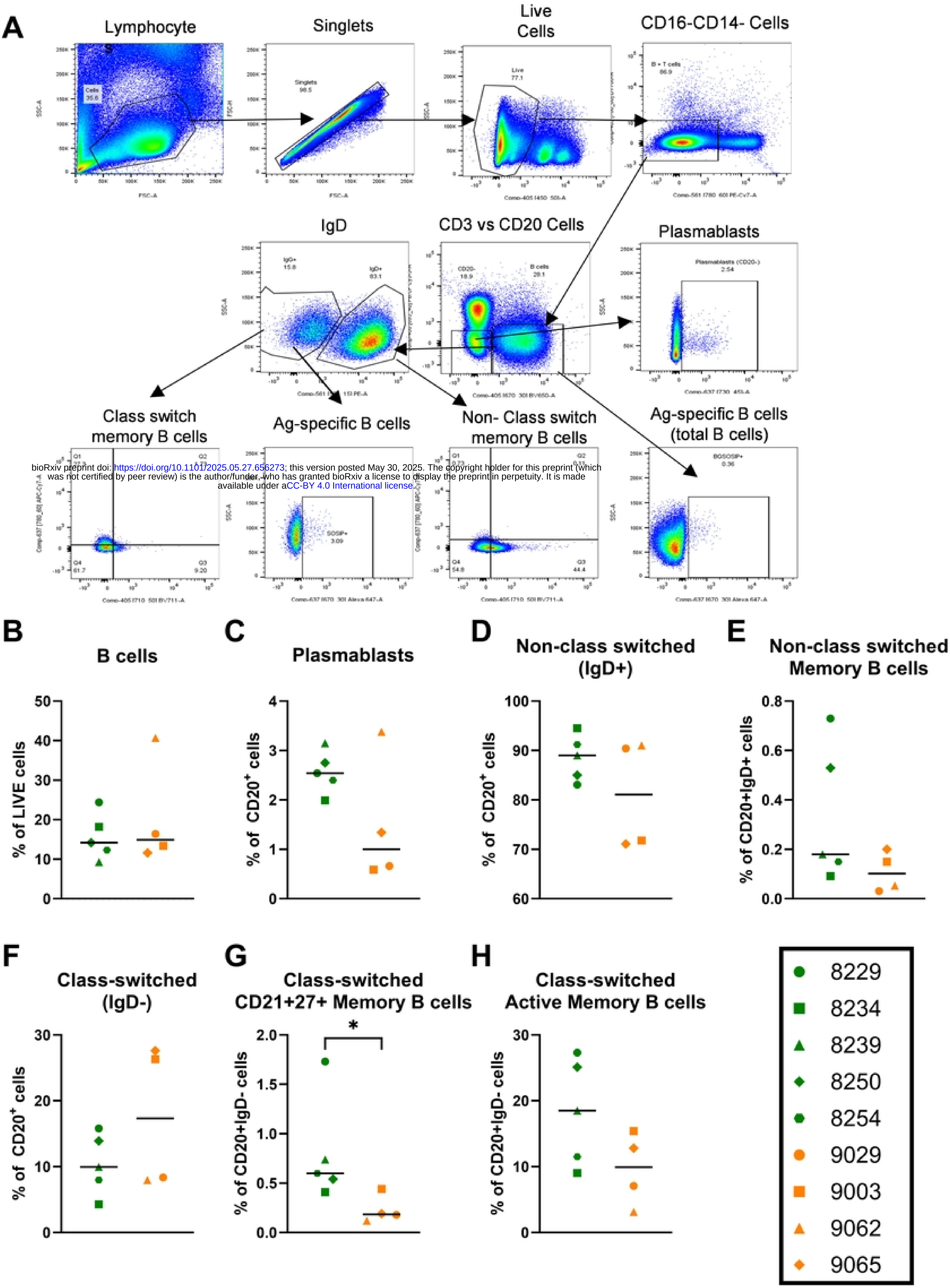

Supp Fig 4
